# Evolution of Conformation, Nanomechanics, and Infrared Nanospectroscopy of Single Amyloid Fibrils Converting into Microcrystals

**DOI:** 10.1002/advs.202002182

**Published:** 2020-12-11

**Authors:** Jozef Adamcik, Francesco Simone Ruggeri, Joshua T. Berryman, Afang Zhang, Tuomas P. J. Knowles, Raffaele Mezzenga

**Affiliations:** ^1^ Department of Health Sciences and Technology ETH Zürich Zürich 8092 Switzerland; ^2^ Department of Chemistry University of Cambridge Lensfield Road Cambridge CB2 1EW UK; ^3^ University of Luxembourg Department of Physics and Materials Science 162a Avenue de la Faïencerie Luxembourg L‐1511 Luxembourg; ^4^ Shanghai University Department of Polymer Materials Nanchen Street 333 Shanghai 200444 China; ^5^ Cavendish Laboratory University of Cambridge J. J. Thomson Avenue Cambridge CB3 0HE UK; ^6^ Department of Materials ETH Zürich Zürich 8093 Switzerland

**Keywords:** amyloid crystals, amyloid fibrils, amyloid polymorphism, nanomechanical properties, secondary structure

## Abstract

Nanomechanical properties of amyloid fibrils and nanocrystals depend on their secondary and quaternary structure, and the geometry of intermolecular hydrogen bonds. Advanced imaging methods based on atomic force microscopy (AFM) have unravelled the morphological and mechanical heterogeneity of amyloids, however a full understanding has been hampered by the limited resolution of conventional spectroscopic methods. Here, it is shown that single molecule nanomechanical mapping and infrared nanospectroscopy (AFM‐IR) in combination with atomistic modelling enable unravelling at the single aggregate scale of the morphological, nanomechanical, chemical, and structural transition from amyloid fibrils to amyloid microcrystals in the hexapeptides, ILQINS, IFQINS, and TFQINS. Different morphologies have different Young's moduli, within 2–6 GPa, with amyloid fibrils exhibiting lower Young's moduli compared to amyloid microcrystals. The origins of this stiffening are unravelled and related to the increased content of intermolecular *β*‐sheet and the increased lengthscale of cooperativity following the transition from twisted fibril to flat nanocrystal. Increased stiffness in Young's moduli is correlated with increased density of intermolecular hydrogen bonding and parallel *β*‐sheet structure, which energetically stabilize crystals over the other polymorphs. These results offer additional evidence for the position of amyloid crystals in the minimum of the protein folding and aggregation landscape.

## Graphical abstract

Atomic force microscopy, infrared nanospectroscopy, and atomistic modelling combined together allow elucidating the molecular origins of the exceptional thermodynamic stability of amyloid crystals, revealing the crucial role of the high density and order of intermolecular *β*‐sheet associated with their purely translational symmetry and lack of mesoscopic twist of amyloid microcrystals.

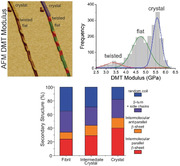

## 1 Introduction

Amyloids are highly ordered structures formed from proteins or peptides, and are associated with a wide range of diseases, including numerous neurodegenerative disorders such as Alzheimer's, Parkinson's, Creutzfeldt‐Jakob diseases, and bovine spongiform encephalopathies.^[^ ^1^, ^2^, ^3^, ^4^, ^5^, ^6^ ^]^ More generally, however, the ability of a large number of peptides and proteins to self‐assemble into amyloid structures opens up the possibility of using these amyloids to develop new nanomaterials for biomedical and nanotechnological applications,^[^ ^7^, ^8^, ^9^ ^]^ and indeed an increasing number of functional roles for natural amyloids are emerging. Therefore, a detailed knowledge of the structural morphology and physical properties of amyloids is of great interest to the scientific community in a very broad context, spanning from medicine to nanotechnology.

The position of amyloid fibrils to emerge as suitable candidates for many technological applications relies on their intrinsic physical properties such as flexibility, mechanical strength, and strong adherence to various substrates. In particular, amyloid structures exhibit remarkable nano‐mechanical properties with Young's moduli in the range of several GPa.^[^ ^10^, ^11^, ^12^ ^]^ Such high values of Young's modulus for amyloids were explained as results of the intermolecular forces between *β*‐sheet and *β*‐layers and as the strong ordering of hydrogen bonds, in which the hydrogen bond network connecting *β*‐strands along the long axis of the fibril has a high cost of free energy to rupture.^[^ ^13^, ^14^, ^15^, ^16^, ^17^ ^]^

The nano‐mechanical properties of amyloids are dependent on the structural morphology.^[^ ^18^, ^19^ ^]^ Short peptide sequences consisting of several amino acids identified from the peptide and protein sequences responsible for the above mentioned diseases possess a tendency to form crystalline structures displaying amyloid‐like properties and the investigation of such microcrystals can provide very important information about the structural morphology of the amyloid core.^[^ ^20^, ^21^, ^22^, ^23^, ^24^, ^25^ ^]^ However, these peptides do not self‐assemble only into one type of structural morphology, rather the self‐assembly process of peptides leads to the formation of various and heterogeneous morphologies including twisted ribbons,^[^ ^26^, ^27^, ^28^, ^29^, ^30^ ^]^ helical ribbons,^[^ ^30^, ^31^, ^32^, ^33^, ^34^ ^]^ nanotubes,^[^ ^35^, ^36^, ^37^, ^38^, ^39^ ^]^ and the already mentioned crystals.^[^ ^20^, ^21^, ^22^, ^23^, ^24^, ^25^, ^40^, ^41^, ^42^, ^43^ ^]^

In the case of self‐assembly of heptapeptide CH_3_CONH‐*β*A*β*AKLVFF‐CONH_2_, the Young's modulus of polymorphic amyloid fibrils was similar although these structures possess different persistence length, and therefore different bending flexibility.^[^ ^30^ ^]^ In such a case, the difference in bending rigidity only occurred as a consequence of the different second area of inertia, that is, with an intrinsically constant Young's modulus. A different situation occurred in the case of self‐assembly of the decapeptide SNNFGAILSS extracted from human islet amyloid polypeptide, where the coexistence of flat ribbons and twisted ribbons was detected and the Young's modulus of flat ribbons was found to be higher compared to twisted ribbons.^[^ ^31^ ^]^ Similarly, a computational study of A*β*(1‐42) amyloid fibrils possessing two different architectures, S‐shaped and U‐shaped, revealed that fibrils with S‐shaped architecture have higher Young's moduli compared to fibrils with U‐shaped architecture.^[^ ^19^ ^]^ Furthermore, the investigation of nanomechanical properties of amyloid crystals self‐assembled from short peptide GNNQQNY also reported significant nanomechanical anisotropy.^[^ ^44^ ^]^ Recently, it has been shown that the short peptides ILQINS, IFQINS, and TFQINS are also able to self‐assemble into amyloid crystals passing through a fibril‐to‐crystal conversion associated with a decrease of free energy in the protein folding landscape.^[^ ^42^ ^]^ It was possible to observe the intermediate crystal, caught in between fibrillar and crystalline states, with flat and twisted regions.

The nanomechanical and morphological properties of amyloid fibrils are furthermore intrinsically related to their secondary and quaternary structure. However, previous studies have mostly only exploited bulk spectroscopic methods to demonstrate that the higher the content of intermolecular *β*‐sheet, the higher the values of Young's modulus tend to be.^[^ ^45^, ^46^, ^47^, ^48^, ^49^ ^]^ The measurement of nanomechanical properties of oligomers, protofibrils and mature fibrils during fibrillization process of *α*‐synuclein and A*β*(1‐42) has revealed an increase of Young's modulus with increase of *β*‐sheet content.^[^ ^45^ ^]^ Short peptides with the sequence SSSSFAFAC self‐assembled at both pH 2 and pH 7 into fibrils with different values of Young's moduli: fibrils at pH 2 with higher *β*‐sheet content had higher Young's modulus compared to fibrils at pH 7 with lower *β*‐sheet content. These results are all consistent with a simple pattern of *β*‐sheet structure increasing the Young's modulus of fibrils when measured at the nano scale.^[^ ^46^ ^]^

While morphological and nanomechanical mapping of amyloid heterogeneity are now routinely applied, most previous studies were limited by the constraint of only using bulk structural methods to correlate these biophysical properties with the secondary structure of the fibrils.^[^ ^50^ ^]^ Bulk spectroscopic methods can only retrieve average information on amyloid polymorphism and do not allow correlation of nanomechanics and secondary structure at the single molecule scale. This challenge has been very recently overcome with the development and application of infrared nanospectroscopy (AFM‐IR) in protein science.^[^ ^49^, ^51^, ^52^ ^]^ The method combines the high‐spatial resolution of AFM with the chemical and structural resolving power of infrared (IR) spectroscopy^[^ ^50^ ^]^ and enables simultaneous correlation at the nanoscale of morphological, chemical, and structural properties.^[^ ^50^ ^]^

In this study we take advantage of the polymorphism of the (I/T)(L/F)QINS hexapeptide model systems and use the single molecule capabilities of peak force quantitative nanomechanical mapping atomic force microscopy (PF‐QNM AFM) and AFM‐IR in combination with atomistic modelling to study and correlate the nanomechanical, chemical, and structural properties of the fibril, intermediate crystal, and crystal forms at the single aggregate scale.

## 2 Results and Discussion

### 2.1 Peak Force Quantitative Nanomechanical Mapping Atomic Force Microscopy (PF‐QNM AFM)

**Figure 1:**
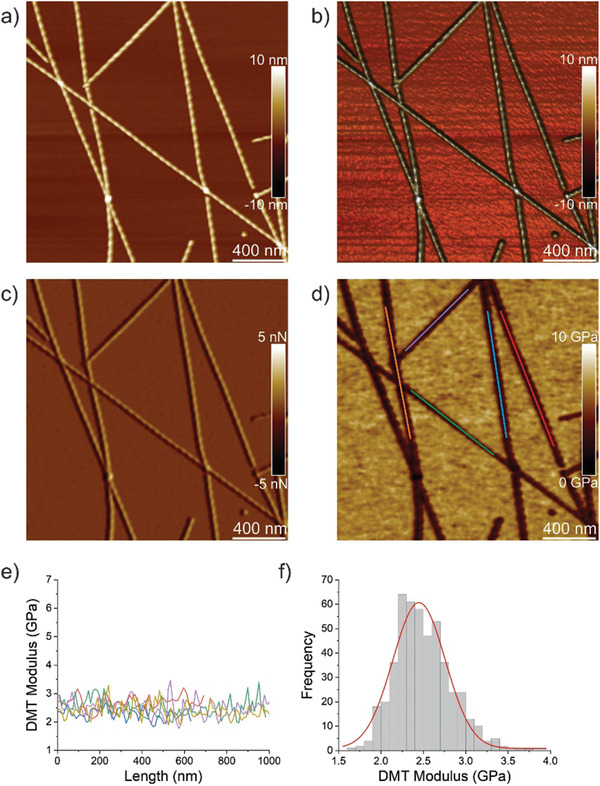
Nanomechanical properties of ILQINS fibrils. a) AFM height, b) 3D AFM height, c) AFM amplitude, and d) AFM DMT modulus of ILQINS fibrils. e) DMT modulus of ILQINS fibrils from part (d). f) The histogram of DMT moduli of ILQINS fibrils.

To probe the differences in nanomechanical and structural properties we first analyzed each peptide individually. ILQINS self‐assembled only into right‐handed ribbons with a regular periodicity around 60 nm; these are displayed in **Figure** **1**a–d and Figure S1b (Supporting Information) is an AFM DMT (Young's modulus using the Derjaguin–Muller–Toporov model) modulus image from which the Young's moduli are extracted and shown in Figure 1e. These values are in range of 2–3 GPa. Figure 1f shows the histogram of the DMT modulus of around 30 different fibrils with Young's moduli of 2.5 ± 0.6 GPa, typical for amyloid fibrils.^[^ ^12^ ^]^

**Figure 2:**
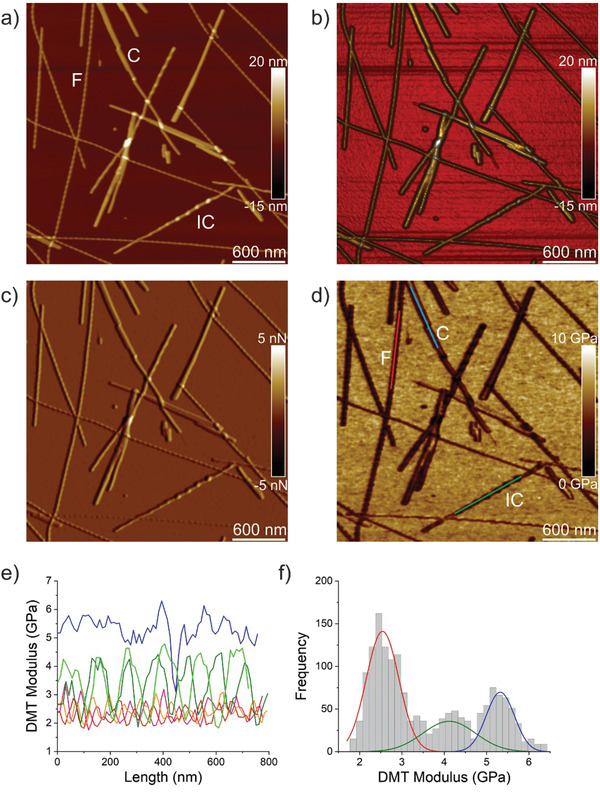
Nanomechanical properties of IFQINS fibrils (F), intermediate crystals (IC), and crystals (C). a) AFM height, b) 3D AFM height, c) AFM amplitude, and d) AFM DMT modulus of IFQINS fibrils. e) DMT modulus of IFQINS fibrils from part (d). f) The histogram of DMT moduli of IFQINS fibrils. Red color corresponds to the DMT moduli of fibrils, green shows intermediate crystals and blue corresponds to the DMT moduli of crystals.

In the case of self‐assembly of IFQINS, the coexistence of fibrils; having structure of right‐handed helical ribbons, right‐handed and left‐handed twisted ribbons; intermediate crystals and crystals was detected, as displayed in **Figure** **2**a–d and Figure S2b (Supporting Information) which shows an AFM DMT modulus image from which the Young's moduli of different structural morphologies were extracted and are shown in Figure 2e. In this case the Young's moduli are different. It is possible to distinguish for each structural morphology a different population of Young's moduli. Fibrils (red color) have Young's moduli in the range 2–3 GPa similar to fibrils self‐assembled from ILQINS. For crystals (blue color) the moduli are in the range 5–6 GPa. Very interesting results were obtained for Young's moduli of intermediate crystals (green color) where the values were spread over 2–5 GPa. The values in the range comparable to that of fibrils were in the twisted part of the intermediate crystal while values close to the range of the crystal were in the flat part of the intermediate crystal. Figure 2f represents the histogram of Young's moduli for all structural morphologies, with approximate ranges of 2.5 ± 0.7 GPa for fibrils, 4.1 ± 1.2 GPa for flat part of intermediate crystals, and 5.3 ± 0.6 GPa for crystals.

**Figure 3:**
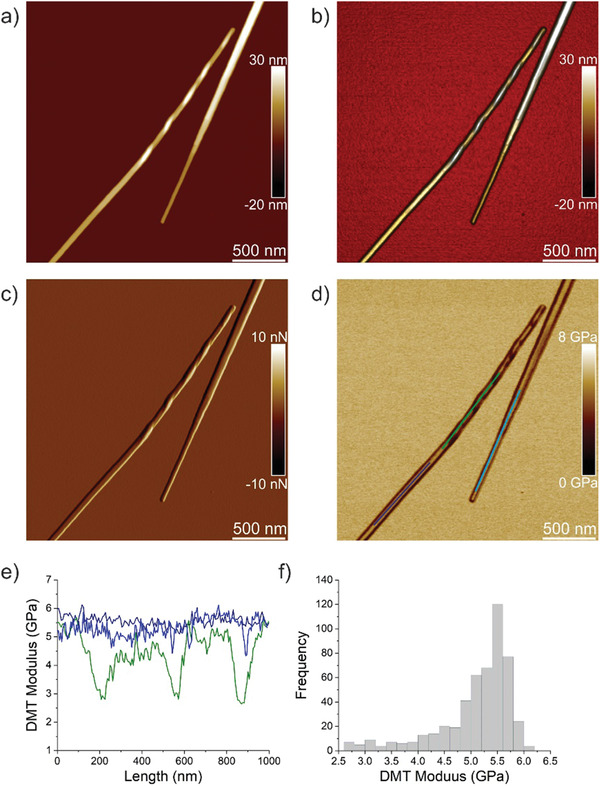
Nanomechanical properties of TFQINS crystals. a) AFM height, b) 3D AFM height, c) AFM amplitude, and d) AFM DMT modulus of TFQINS crystals. e) DMT modulus of TFQINS crystals from part (d). f) The histogram of DMT moduli of TFQINS crystals from part (e).

The peptide TFQINS self‐assembled mainly into microcrystals together with a small amount of twisted ribbons.^[^ ^42^ ^]^ However, in the case of TFQINS a very clean fibril‐to‐crystal conversion can be observed (**Figure** **3**a–c) therefore the change of Young's modulus on conversion was analyzed within the same single fibril. The height profiles of microcrystals are displayed on Figure S3 (Supporting Information). Figure 3d shows an AFM DMT modulus image of crystal and fibril‐to‐crystal conversion from which the values of Young's moduli were extracted (Figure 3e). Crystals (blue color) have Young's moduli in the range 5–6 GPa similar to crystals self‐assembled from IFQINS. Fibril‐to‐crystal conversion (green color) has values in the range of 3–6 GPa (Figure 3f). Thus, there is a similar trend as in the case of IFQINS, where the twisted part of fibril‐to‐crystal conversion has a lower Young's modulus compared to the flat part.

**Figure 4:**
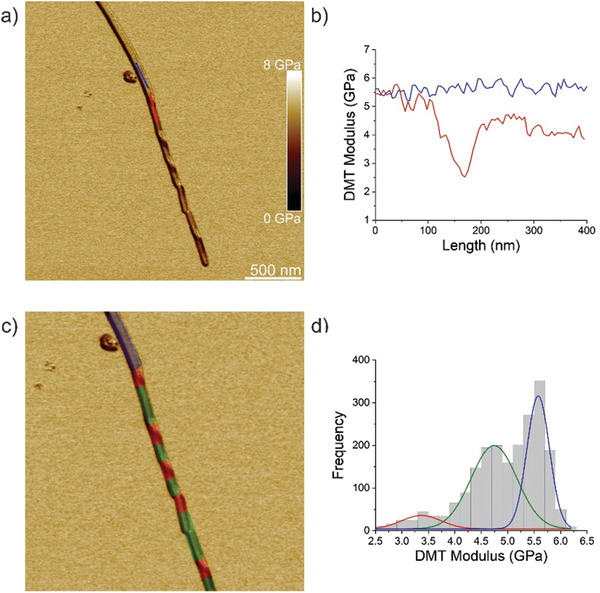
Nanomechanical properties of TFQINS fibril/crystal transition. a) AFM DMT modulus of TFQINS crystal. b) DMT modulus of the TFQINS crystal from part (a). c) AFM DMT modulus of TFQINS crystal tinted red, green or blue by the assigned region. d) The histogram of DMT moduli of TFQINS crystals. Red and green colors indicate twisted or flat sections of the fibril/crystal transition region, respectively; blue corresponds to the crystal region.

**Figure** **4** summarizes results from a detailed analysis of the Young's moduli of a fibril‐to‐crystal conversion. Figure 4a shows the AFM DMT moduli image of an individual fibril‐to‐crystal conversion with corresponding values of Young's moduli displayed in Figure 4b. The Young's modulus of the crystal part (blue color) is nearly constant around 5.5 GPa. In the twisted part the value decreases and increases again in the flat part of the conversion region. However, the modulus does not return to values of the crystal part, shown by the gap between the red and blue lines in Figure 4b. Analysis of the fibril‐crystal transition in even greater detail is displayed in Figure 4c where red tint corresponds to twisted parts, green to flat parts of the conversion region, and blue to the crystal part. Figure 4d represents the histogram of Young's moduli of TFQINS crystals and fibril‐to‐crystal conversions with values of 3.3 ± 0.8 GPa for twisted part, 4.2 ± 1.0 GPa for flat part of conversion and 5.6 ± 0.5 GPa for crystal.

### 2.2 Infrared Nanospectroscopy (AFM‐IR)

**Figure 5:**
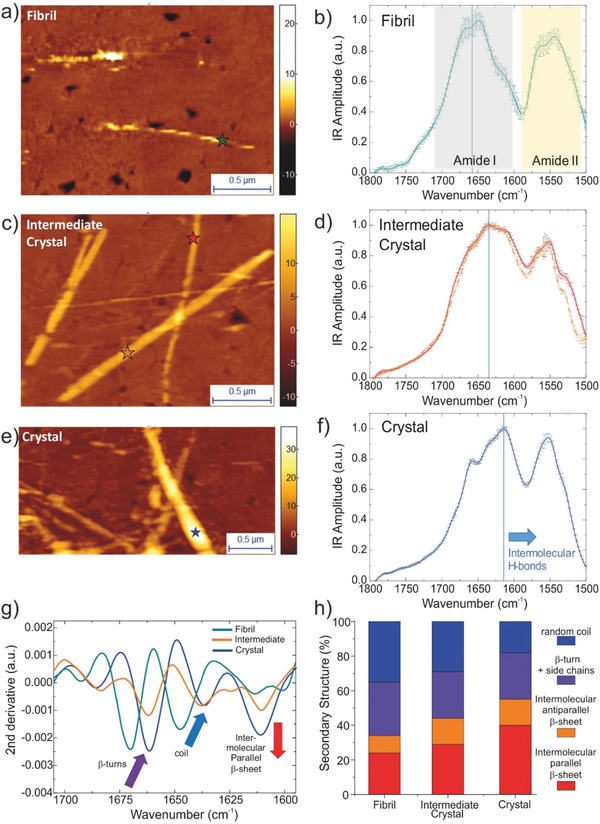
AFM‐IR of IFQINS fibril, intermediate crystal, and crystal. Morphology and nanoscale localized IR absorption spectra in the Amide I and II bands of a, b) periodic fibril, c, d) intermediate crystal, and e, f) crystal. g) Second derivative analysis of the Amide band I to deconvolve secondary structural contribution. h) Quantification of percentage of secondary structure by integration of their spectra.^[^ ^54^, ^55^, ^56^ ^]^ The structural contributions were assigned from spectroscopic data in literature.

To provide further insight into the correlation between the nanomechanical properties and the chemical and secondary structure heterogeneity of single fibrils and crystals, we applied infrared nanospectroscopy (**Figure** **5** and Figure S4, Supporting Information). We selected the IFQINS peptide for examination by the AFM‐IR tool since it is the most heterogeneous sample (Figure 2).

AFM‐IR permitted characterization within the same sample of the chemical and structural heterogeneity of single aggregates at the nanoscale. The morphology maps acquired by AFM‐IR showed the coexistence of twisted fibrils (Figure 5a) and crystals (Figure 5c,e). Following the acquired morphology maps, it was possible to pinpoint the probe of the AFM‐IR system to acquire nanoscale resolved IR spectra (Figure 5b,f) in the spectroscopic region of the amide bands I and II generally used as the “fingerprints” of proteins and peptides, for each of the different morphological types, with a lateral spatial resolution in the order of ≈10 nm.^[^ ^53^ ^]^

In the observed spectra, as expected for short peptides, the Amide band I has similar or lower intensity than the Amide band II.^[^ ^54^ ^]^ Since the Amide I band arises from the stretching vibration of the C=O of the protein backbone, it is intimately connected to the secondary and quaternary conformation, enabling quantitative structural determination. The AFM‐IR analysis of the structural heterogeneity within the heterogeneous IFQINS sample allowed the absolute determination and comparison of the secondary structure of the fibrillar and crystal states, separate to signal from the chemical variation due to sequence. Indeed the measurements of peptides with different primary sequence (ILQINS, IFQINS, and TFQINS) could affect a fine determination of the secondary structure, since the residual IR absorption of amino acid side chains of protein can contribute up to ≈10% to the infrared absorption arising from the Amide I band of protein.^[^ ^55^ ^]^

The IR spectra showed a strong shift of the Amide I band to lower wavenumbers when passing from the twisted fibril state (≈1648 cm^−1^, 2.5 ± 0.7 GPa) (Figure 5b), to the crystal state. In particular, we could distinguish two classes of spectra of the crystal. The first with position of the peak of the Amide I band closer to the one of the fibrils (≈1630 cm^−1^) (Figure 5d), and the second with Amide band I peak at lower wavenumber (≈1615 cm^−1^) (Figure 5f). The shift to lower wavenumber of the Amide I band is caused by an increase of intermolecular parallel *β*‐sheet structure and of the hydrogen bonding network,^[^ ^56^ ^]^ thus we could assign the first class of spectra to the intermediate crystal state (4.1 ± 1.2 GPa) and the second class to the crystal state (5.3 ± 0.6 GPa).

To investigate the subtle structural changes observed upon conversion from the fibrillar to the intermediate crystal and crystal state, we de‐convolved the structural contributions in the Amide band I by second derivative analysis (Figure 5g).^[^ ^50^, ^57^, ^58^ ^]^ From the fibrillar to the intermediate crystal, we observed a net increase of intermolecular parallel *β*‐sheet content (red arrow, ≈1610 cm^−1^) and slightly of antiparallel *β*‐sheet conformation (≈1685–1695 cm^−1^), versus a net decrease of random coil conformation (blue arrow, 1648→1638 cm^−1^) (Figure 5g). Then, we observed a further increase of intermolecular *β*‐sheet content from the intermediate to the crystal state. The shift of random coil, *β*‐turn and side chains contribution to lower wavenumbers is related to the general shift of the Amide band I at lower wavenumbers because of the more collective, longer‐range hydrogen bonding (Figure 5g). On the other hand, the shift of the antiparallel *β*‐sheet band at lower wavenumbers suggests a conformational rearrangement from the fibril to the crystal structure. Overall, from the fibril to the crystal state, we observed a total increase of intermolecular *β*‐sheet of +21 ± 5% and a decrease of random coil conformation of −17 ± 5% (Figure 5h). We did not observe a significant variation of *β*‐turn conformation. The secondary structure of the crystal state was composed of 55% intermolecular *β*‐sheet and 27% *β*‐turns, related to the intermolecular interactions of the peptides, and only a residual 18% of disordered random coil conformation.

These results can be related to the increase of Young's modulus from the fibril (2.5 ± 0.7 GPa) to crystal states (5.3 ± 0.6 GPa). The increase of intermolecular *β*‐sheet content is related to an increased number of contacts in the network of intermolecular hydrogen bonds, as demonstrated by the downward shift of the weight of the whole spectrum. The longer‐range ordering of the *β*‐sheet hydrogen bond network increases the zero‐frequency stiffness of the material. Thus, the IR‐spectroscopy and AFM indentation together are able to show both sides of the atomic‐scale organization and material‐scale zero‐frequency stiffening.

### 2.3 Atomistic Modelling

Atomistic simulations of the indentation process allow us to further interrogate the changes in amyloid material properties following the gain of crystal‐like order. Taking advantage of the parallel *β*‐sheet atomistic structures available for the ILQINS peptide,^[^ ^42^ ^]^ small aggregates having this sequence (which prefers twisted fibrils in solution more than nanocrystals)^[^ ^42^ ^]^ were chosen for study in simulation.

The ILQINS aggregate structures were introduced unchanged from a previous work,^[^ ^24^ ^]^ except that the number of peptides along each axis of the initial system was varied so as to give a “thick” and “thin” model system, serving as proxies for the “nanocrystal” and “fibril” physical systems (see Experimental Section).

**Figure 6:**
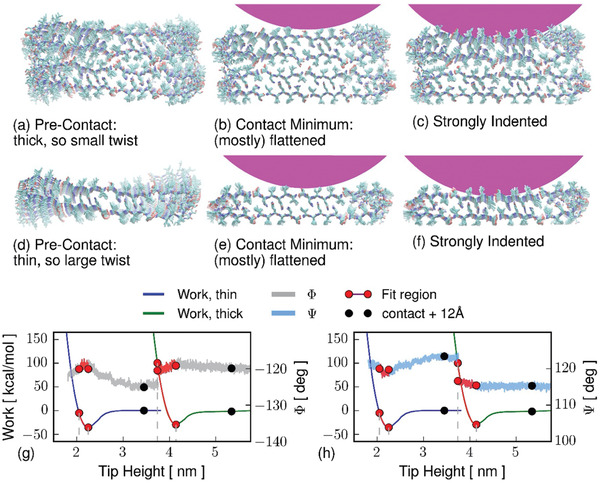
Atomistic simulation of indentation of a–c) thick and d–f) thin amyloid models. Due to the smaller aspect ratio, the thicker system is less twisted at initial contact, and so has less opportunity to relax under indentation. The equilibrium work is shown as smooth curves on the left axes, the same data is repeated on (g, h) for separate comparison against each Ramachandran angle. Averages of each Ramachandran angles were taken over the 120 residues nearest to the AFM tip center (g:Φ angle, h:Ψ). Circles correspond to images in part (a–f) and also to Ramachandran histograms in Figure 7. Pink circle caps indicate the tip; direction of indentation is down the page; the fibril axis points out of the page.

The initial relaxation of the structures before indentation agrees with our previous work showing that the thickness of the sample is related to the mesoscopic polymorphism:^[^ ^24^, ^42^ ^]^ the thin structure does not build up significant internal shear when twisting, so is twisted even on the surface. The thicker structure cannot twist without shearing, so it is near‐rectangular on the surface (**Figure** **6**a,d). We identify the twisted and near‐rectangular structures respectively to the fibrils and nanocrystals observed in vitro^[^ ^42^ ^]^ and previously shown to have locally very similar atomistic configurations.

**Figure 7:**
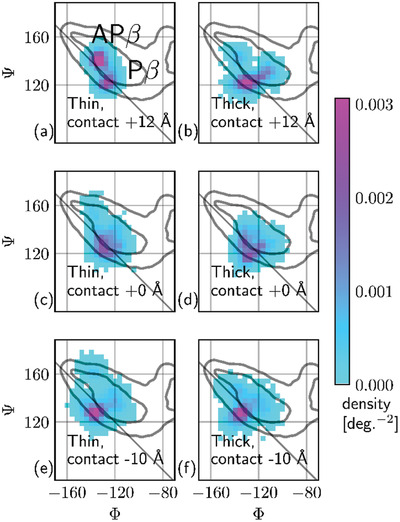
Ramachandran histograms taken over the 120 residues nearest to the tip during the atomistic simulations, at 12 ns (equals 12 Å) before the contact minimum, at the minimum, and 10 Å past the minimum. The diagonal line indicates Φ = −Ψ: structures lying on this line have no strand twist. Structures closer to the upper‐left corner are more extended. Before being flattened to the surface, the thin (fibril‐like, on the left) structure has extended backbone conformation in some residues. At contact it takes on a more pleated structure with less twist. Under extreme indentation the two samples have similar compact and non‐twisted conformations. The thick (nanocrystal‐like, on the right) structure begins as compact and remains so, changing much less under indentation.

The twisting/untwisting of the thin fibril‐like structure is related to backbone configuration. The thin structure has a less compact backbone than the thick structure, having in its free state a certain amount of extended (AP‐like) backbone conformation which is relaxed to a more compact state as the fibril is pushed onto the surface, and which then extends again (slightly) as the indentation increases (Figures 6 and **7**, and Figure S5, Supporting Information). From the fitted regions shown in red on Figure 6 we recover Young's moduli of *E* = 35.3 GPa and *E* = 38.9 GPa for the fibril and nanocrystal proxy systems respectively, roughly eight times the experimental values. Differences of one order of magnitude between experimental Young moduli in amyloid systems and MD simulations are typical and have been reported by several authors before,^[^ ^11^, ^59^, ^60^ ^]^ a possible explanation is an over‐hard treatment of the Pauli exclusion force in conventional force fields, which the newest developments in simulation technology are beginning to address.^[^ ^61^ ^]^ The simulations do agree however, that the Young's moduli of crystals exceed by 3.6 GPa those of twisted amyloids, showing the same ordering of crystal > fibril as in the experimental indentation.

By plotting backbone dihedral angles in Figure 6 we are able to see that indentation is accompanied by significant conformational change, shown as histograms on the Ramachandran plane in Figure 7. The thin fibril‐like structure loses some of its AP‐like conformation under pressure, as it untwists and is flattened to the surface it becomes more like the thicker crystal‐like system, with classical P‐*β* Ramachandran angles. The thicker, less‐twisted, structure remains in the P‐*β* region, only changing by becoming slightly more disordered on contact with the tip. The linear‐seeming unit‐cell shape changes of shortening and un‐twisting (pleating) which accompany the global transition from a twisted to a flat shape, are reflected at the atomistic level by the loss of AP‐like extended backbone conformations. Residues occupying AP‐like conformations are scattered along the peptide chains but have a cooperative order along the fibril hydrogen bonding axis (Figure S5, Supporting Information).

### 2.4 Results Overview

Indentation using AFM interrogates stiffness to large deformations at low frequency (“thermodynamic regime”), while IR accesses the stiffness of individual vibrational modes (“vibrational regime”), measuring near‐equilibrium excitations in the femtosecond range. The driving quantities for a high Young's modulus are density of contacts, strength of contacts, and collective ordering of contacts over the tip lengthscale interrogated, while the IR accesses both short length scales and the larger scales related to hydrogen bonding cooperativity.

Taken together, the reported data indicate that the fibril‐to‐crystal transition in amyloid is associated with an increase in intermolecular pleated *β*‐sheet and hydrogen bonding, which results in overall shift of the Amide I band to lower vibrational frequencies. The shift to longer wavelengths is favorable for stabilization of the crystal structure via the vibrational entropy and via the long‐range order of hydrogen bonds. The fibril structure has a stiff network of strong hydrogen bonds. The fibril is soft to indentation because it can untwist and flatten without losing its hydrogen bond network: the hydrogen bonds rather become more collective and more ordered over a long range with the loss of twist. The emerging picture clearly supports an overall gain in free energy via a fibril‐to‐crystal transition, contributing to the exceptional stability of amyloid‐like crystals, consistent with the postulated lower position of the crystals in the energy landscape compared to all other amyloid polymorphs.^[^ ^39^, ^42^ ^]^

## 3 Conclusion

We have used a combination of single molecule atomic force microscopy imaging, nano‐indentation and nanoscale chemical spectroscopy in combination with atomistic modelling to unravel and directly correlate the nanomechanical and vibrational properties of amyloid polymorphs, including fibrils and microcrystals, on a homologue series of hexapeptides fragments from lysozyme (ILQINS, IFQINS, and TFQINS). Specifically, we have been able to relate mesoscopic structures of the polymorphs to the secondary and quaternary structures of the hexapeptides and to the density of intermolecular hydrogen bonds. We have shown that amyloid fibrils and microcrystals have different Young's moduli, varying in the range of 2–6 GPa, with amyloid crystals having systematically larger Young's moduli. The origin of this increase in Young's moduli arises from the higher density and order of intermolecular *β*‐sheet and increased lengthscale of cooperativity in the microcrystal structures, which possess purely translational symmetry, as opposed to twisted fibril structures. This is evidenced by both the infrared nanospectroscopy vibrational spectra and the Ramachandran angles of the hexapeptides as a function of the overall fibril twist. Remarkably, the correlation between Young's modulus and vibrational spectra can be resolved even for events where a single amyloid fibril untwists to undergo a fibril‐crystal transition, providing an unprecedented and compelling map of the mesoscopic, atomistic, and vibrational properties along one and the same amyloid aggregate, and elucidating the molecular origins of the exceptional thermodynamic stability of amyloid crystals. We are able to support the use of linear elastic theories of biomolecule assembly, by connecting the highly non‐linear atomic dynamics of peptides to a relatively smooth and linear‐seeming mechanical response on nanometer scales.

## 4 Experimental Section

##### Peptide Synthesis

The hexapeptides ILQINS, IFQINS, and TFQINS were synthetized by standard solid phase peptide synthesis as described in previous works.^[^ ^24^, ^42^ ^]^

##### Self‐Assembly of Hexapeptides

The lyophilized hexapeptides ILQINS, IFQINS, and TFQINS were mixed with Milli‐Q water at concentration of 5 mm and left self‐assemble at room temperature without stirring for 24 h.

##### Peak Force Quantitative Nanomechanical Mapping Atomic Force Microscopy (PF‐QNM AFM)

A droplet of 20 mL of solution of ILQINS, IFQINS, and TFQINS was deposited on freshly cleaved mica, incubated for 2 min, rinsed with Milli‐Q water, and dried by a pressurized air. PF‐QNM AFM was carried out using a Nanoscope VIII Multimode Scanning Force Microscope (Bruker, USA) operating at ambient conditions and covered by an acoustic hood to minimize vibrational noise. The cantilever (Bruker, USA) was calibrated as described in a previous work.^[^ ^62^ ^]^ Images were flattened and analyzed using the NanoScope Analysis 8.15 software and FiberApp software.^[^ ^63^ ^]^

##### Infrared Nanospectroscopy (AFM‐IR)

An aliquot of 10 µL of the sample was deposited on the flat gold surface for 20 s, to reduce mass transport phenomena during drying. Successively, the droplet was rinsed by 1 mL of Milli‐Q water and dried by a gentle stream of nitrogen.

A nanoIR2 platform (Anasys, USA) with a tunable quantum cascade laser (QCL) with top illumination configuration was used. Sample morphology was scanned with a rate line within 0.1–0.4 Hz and in contact mode. A silicon gold coated PR‐EX‐nIR2 (Anasys, USA) cantilever with a nominal radius of 30 nm and an elastic constant of about 0.2 N m^−1^ was used. The IR light was polarized perpendicular to the surface of deposition to exploit field enhancement^[^ ^49^ ^]^ between the gold coated tip and the gold substrate. Both spectra and images were acquired by using phase loop (PLL) tracking of contact resonance, the phase was set to zero to the desired off‐resonant frequency on the left of the IR amplitude maximum, and tracked with an integral gain *I* = 0.1 and proportional gain *P* = 5.^[^ ^51^, ^56^, ^58^ ^]^ All images were acquired with a resolution of at least 300 × 300 pixels per line.

The AFM images were treated and analyzed using SPIP software. The height images were first order flattened, while IR and stiffness related maps where only flattened by a zero‐order algorithm (offset). Nanoscale localized spectra were collected by placing the AFM tip on the top of the fibril with a laser wavelength sampling of 2 cm^−1^ and 256 co‐averages, within the range 1400–1800 cm^−1^.

Within a fibril, spectra where acquired at a least ten spectra in at least two different nanoscale localized positions. Successively, the spectra were smoothed by FFT (3 pts) and Savitzky–Golay filter (second order, 11 points), normalized and averaged. Spectra second derivatives were calculated, smoothed by Savitzky–Golay filter (second order, 11 points). Relative secondary and quaternary organization was evaluated integrating the area of the different secondary structural contribution in the amide band I, as previously shown in literature.^[^ ^50^, ^57^, ^64^ ^]^ The error in the determination of the relative secondary structure content is calculated over the average of at least five independent spectra and it is <10%. Spectra were analyzed using the microscope's built‐in Analysis Studio (Anasys) and OriginPro. All measurements were performed at room temperature and with laser power between 1–15% of the maximum and under controlled Nitrogen atmosphere with residual real humidity below 5%.

##### Model Setup

Two model amyloid structures were prepared from the atomistic unit cell structures previously reported.^[^ ^24^ ^]^ Both assemblies were of size 4 peptides (≈80 Å) in the peptide backbone axis direction (*a* lattice axis) and 24 peptides (≈116 Å) in the hydrogen bonding axis direction (*c* lattice axis). The “thin” model was 4 peptides thick (≈20 Å) in the sidechain interdigitation (*b* lattice axis) direction, while the “thick” model was 8 peptides (≈35 Å) thick. Both models were simulated in vacuum for 10 ns and then allowed to relax onto a graphene monolayer of 8262 atoms. The monolayer was artificially stiffened with Cartesian restraints in order to imitate the effect of a thicker surface, preventing the graphene sheet from flexing or buckling. The AFM tip was modelled with the cap of a Fibonnaci spherical shell of radius of curvature 80 Å, with 336 Si atoms held rigid relative to each other on the surface of the cap. The pmemd simulation program^[^ ^65^, ^66^ ^]^ was used together with the AMBER 15‐ipq‐vacuum^[^ ^67^ ^]^ force field.

##### Indentation Simulation Methods

The simulated Silicon tip was lowered into the sample at a fixed speed of 0.1 ms^−1^, to a depth of about 15 Å below the contact minimum or 2 nm below first contact (simulation time therefore about 20 ns per indentation). The indentation depth of 2 nm was noted as the point at which forces became very large. To reduce artifacts due to the relatively fast indentation speed required, a Jarzynski calculation^[^ ^68^ ^]^ was made in which (eight) replicate simulations were carried out for each system and the equilibrium work *W* estimated from the nonequilibrium work values *W*’ as exp( − *βW*) = 〈exp( − *βW*′)〉. Young's modulus is problematic to extract for thin samples as multiple parameters need to be estimated or fit, however a standard procedure which is qualitatively accurate is to fit a Hertzian model to the indentation process.^[^ ^69^ ^]^ This procedure assumes no effects of the sample thickness or underlying surface, and assumes constant contact area during the indentation. The Poisson ratio was set to *η* = 0.5 (typical for biological samples) and the contact area was estimated at A = 4000 Å^2^. Reference contours of the Ramachandran plots were taken from the Duke dataset.^[^ ^70^ ^]^ Ramachandran angles were collected using cpptraj.^[^ ^71^ ^]^

## Conflict of Interest

The authors declare no conflict of interest.

## Supporting information

Supporting Information
