# Supplementary material for: Evolution of Conformation, Nanomechanics, and Infrared Nanospectroscopy of Single Amyloid Fibrils Converting into Microcrystals
Source: Adv Sci (Weinh). 2020 Dec 11;8(2):2002182. doi: 10.1002/advs.202002182 (PMC7816722; doi:10.1002/advs.202002182)
Supplement: Supplementary file 1 — Supporting Information [file ADVS-8-2002182-s001.pdf]

© 2020 Wiley-VCH GmbH

ADVANCED SCIENCE Open Access

Supporting Information

for *Adv. Sci.*, DOI: 10.1002/advs.202002182

Evolution of Conformation, Nanomechanics and Infrared Nanospectroscopy of Single Amyloid Fibrils Converting into Microcrystals

*Jozef Adamcik, Francesco Simone Ruggeri, Joshua T. Berryman, Afang Zhang, Tuomas P. J. Knowles and Raffaele Mezzenga**

© 2020 Wiley-VCH GmbH

Supporting Information

Evolution of Conformation, Nanomechanics and Infrared Nanospectroscopy of Single Amyloid Fibrils Converting into Microcrystals

*Jozef Adamcik, Francesco Simone Ruggeri, Joshua T. Berryman, Afang Zhang, Tuomas P. J. Knowles and Raffaele Mezzenga**

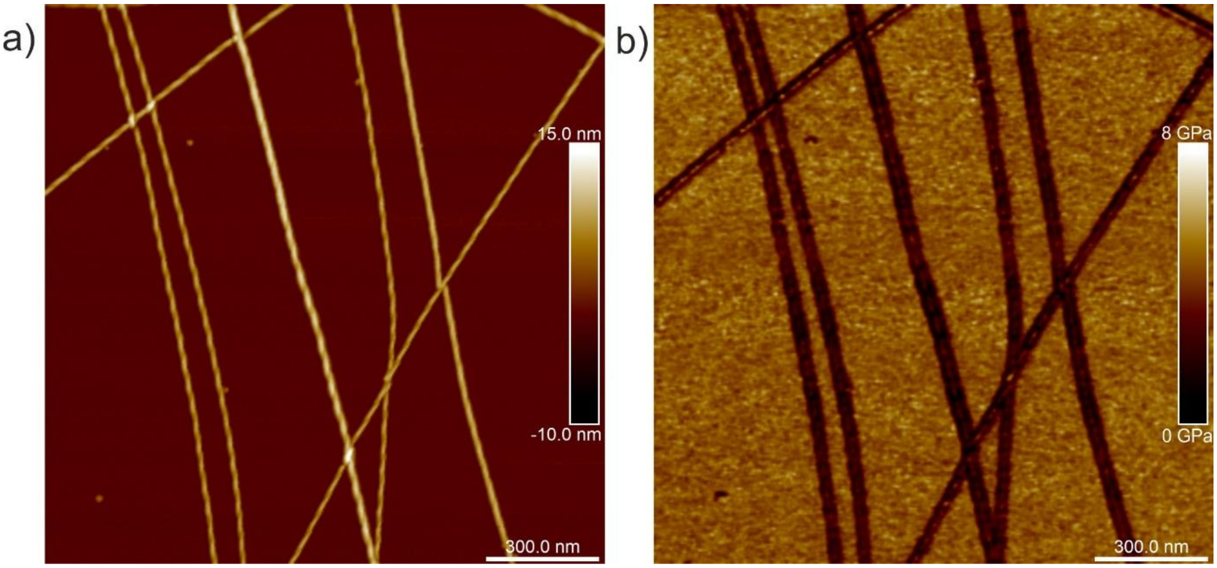

Figure S1. a) AFM height and b) AFM DMT Modulus of ILQINS fibrils.

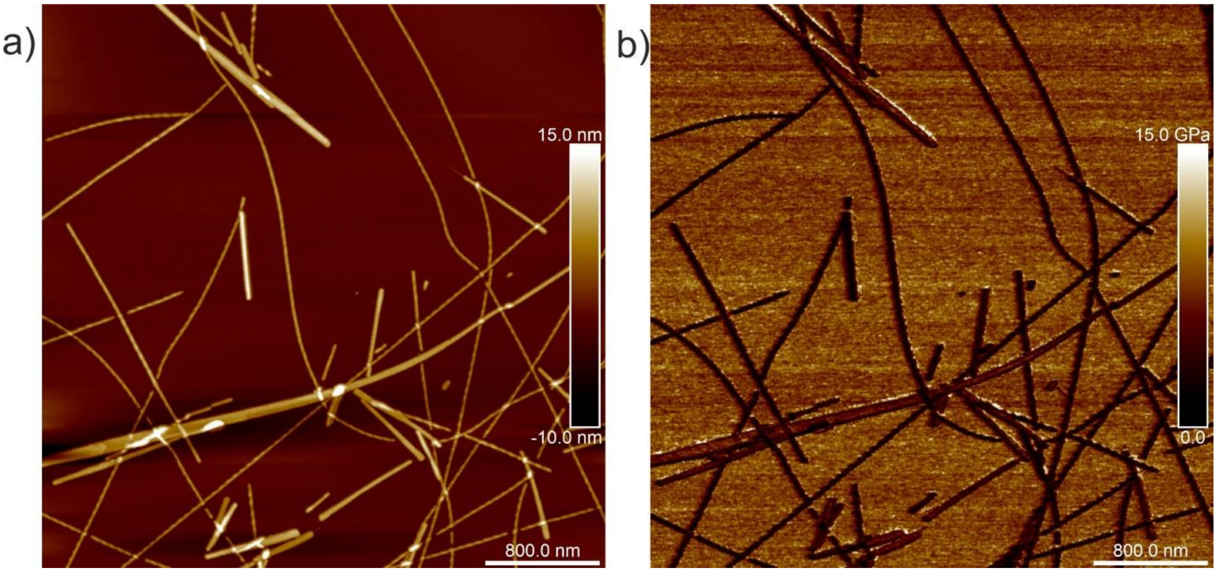

Figure S2. a) AFM height and b) AFM DMT Modulus of IFQINS fibrils.

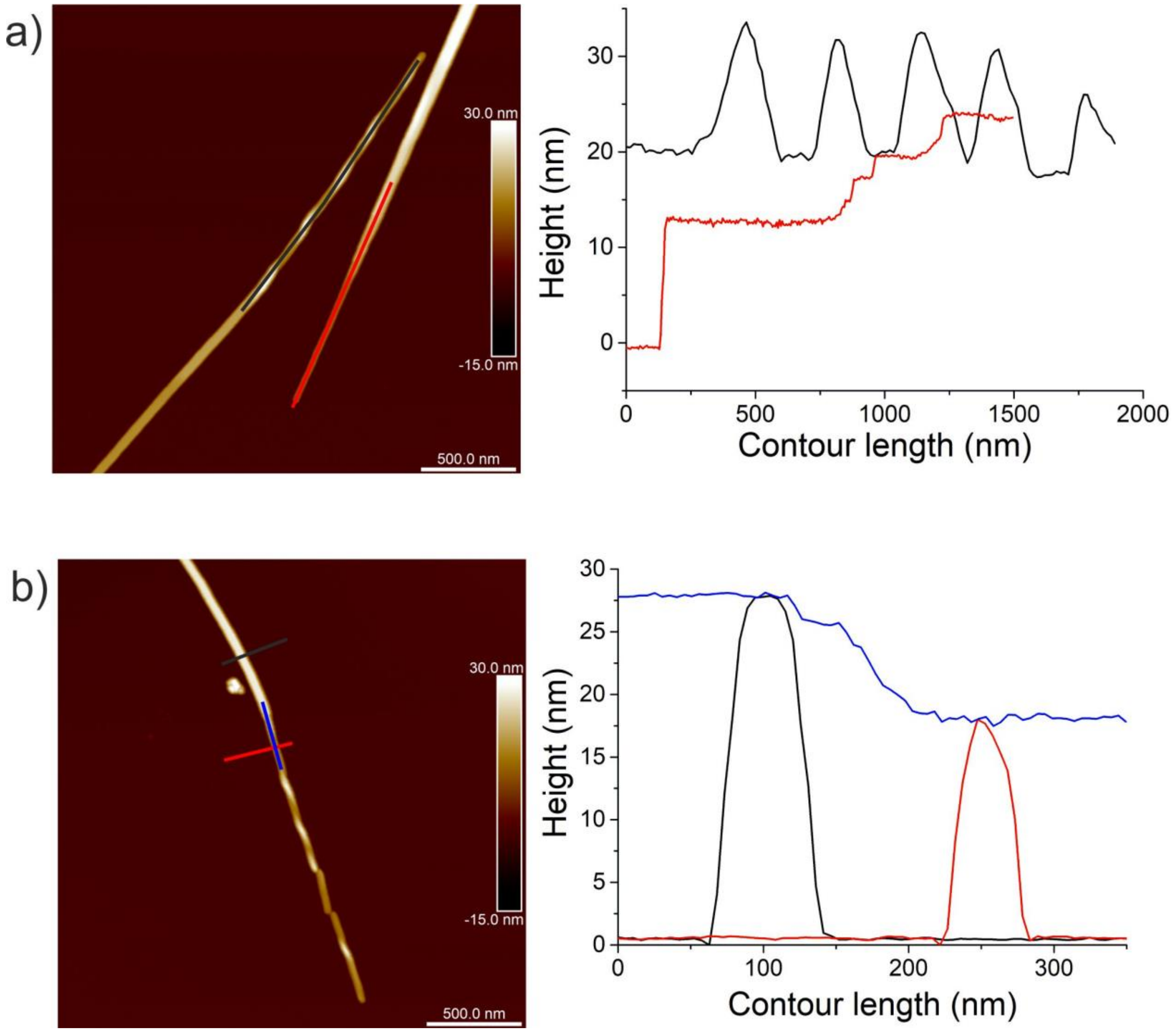

Figure S3. Structural morphology of TFQINS crystals. a) AFM height with corresponding longitudinal (black and red color) height profiles of TFQINS crystals. b) AFM height with corresponding transversal (black and red color) and longitudinal (blue) height profiles of TFQINS crystal.

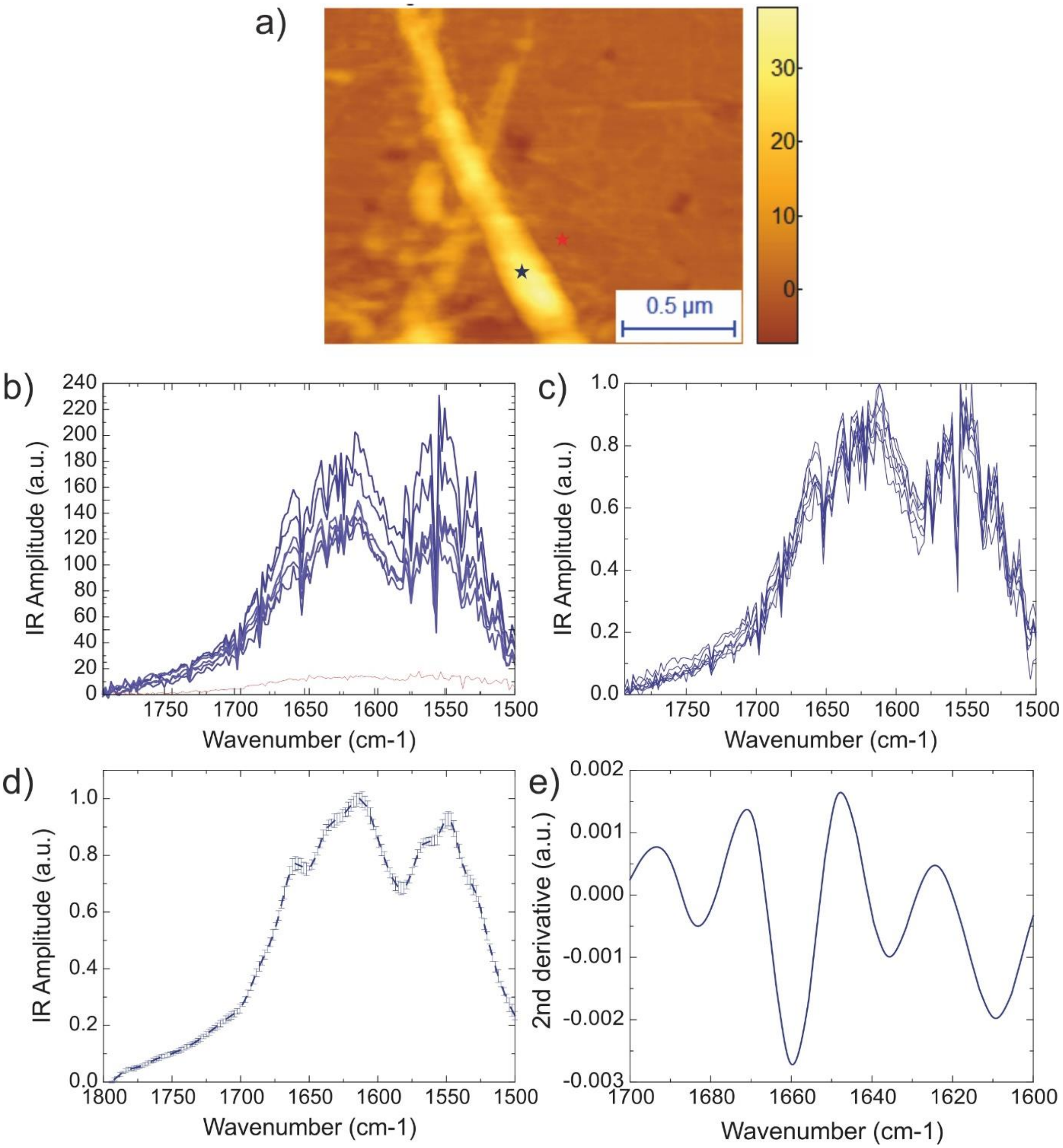

Figure S4. Method of AFM-IR analysis. a) At a position on a single fibril or crystal, at least 5 spectra are acquired with high signal to noise ratio (blue star), where the noise is represented by a spectrum acquired at a position close to the fibril on the substrate (red star). Spectra are then b) baselined and normalized to 1, c) averaged smoothed and de-spiked first by a Fast Fourier Transform filter (FFT, 3 pt) and then by a Savitzky-Golay filter (SG, 2nd order 11 pts) (avg+SD). e) To evaluate the secondary structure the second derivative of the average spectrum in the Amide Band I is calculated, smoothed (SG 2nd order 11 pts) and then the de-convoluted structural contributions are integrated.

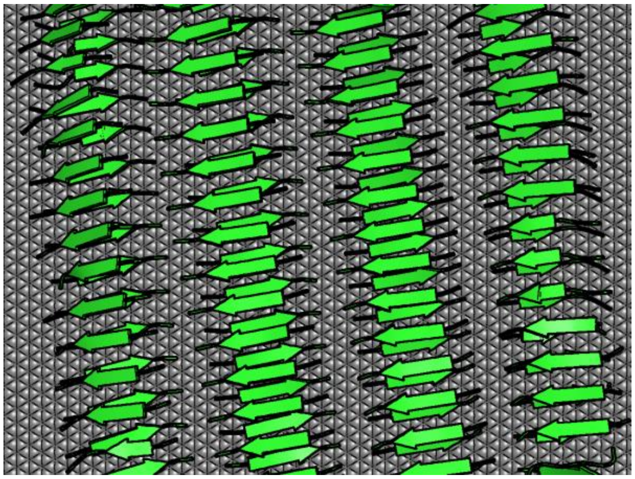<br>Thin, t=-10ns.	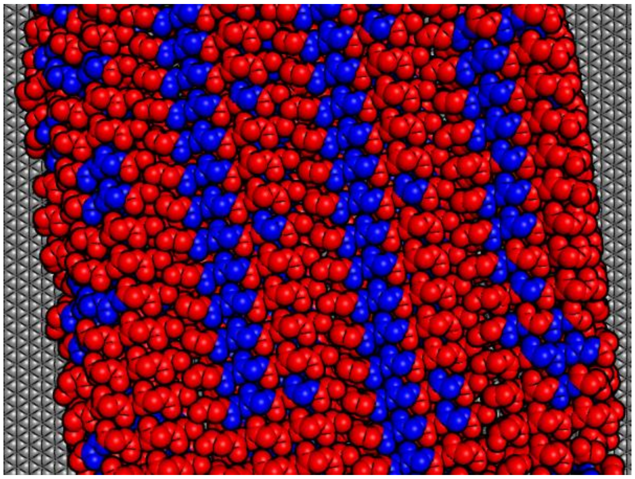
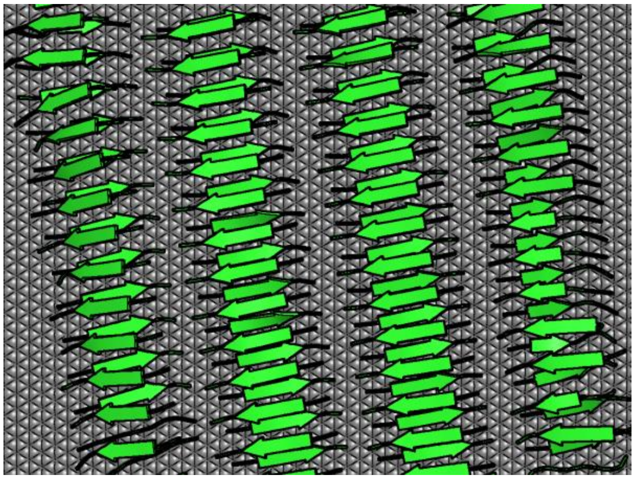<br>Thin, t=+12ns.	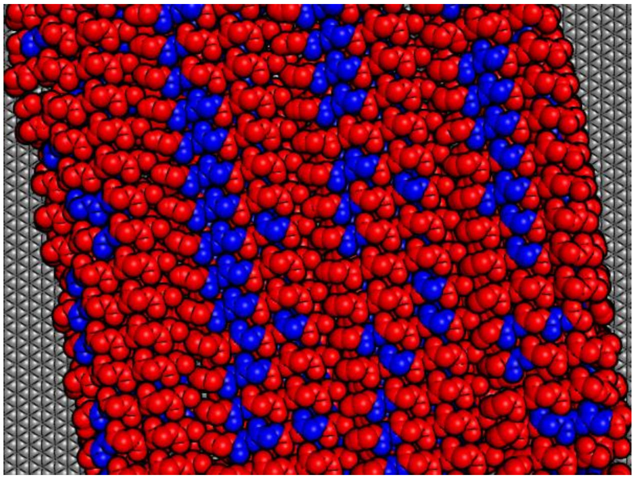
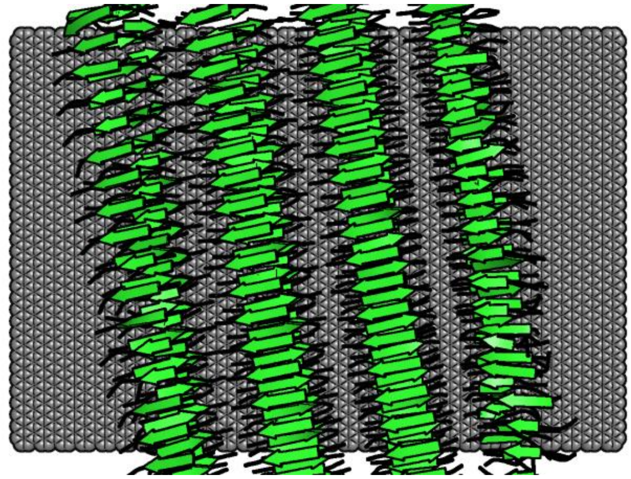<br>Thick, t=-10ns.	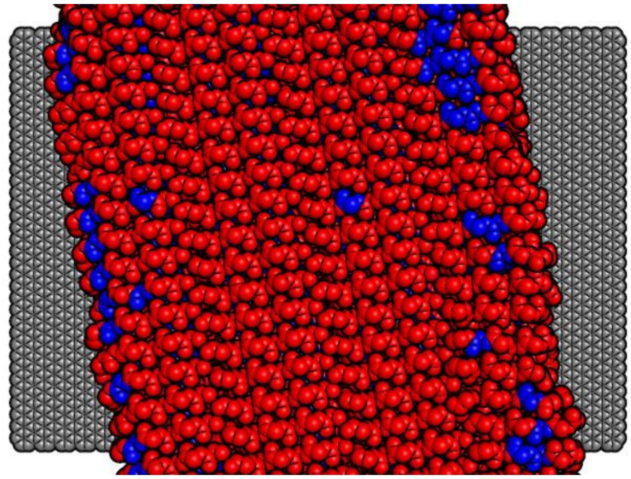

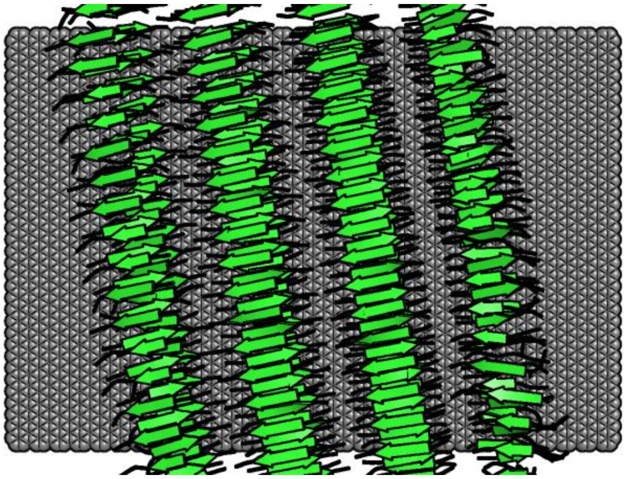<br>Thick, t=+12ns.	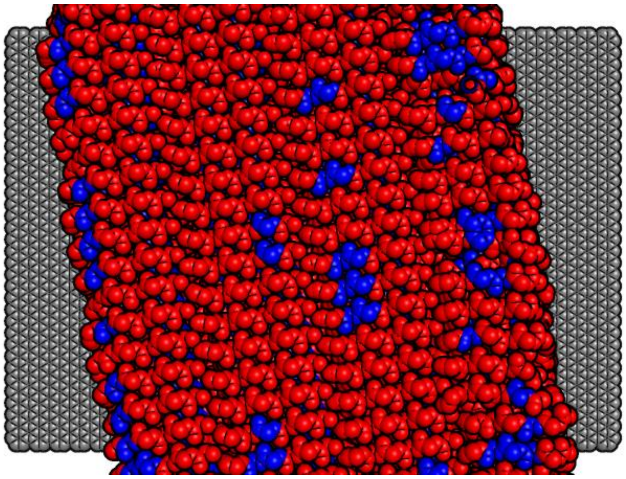

Figure S5. Table of snapshots, showing P beta sheet order (left column), and backbone conformation consistent with P or AP beta sheet (right column). Rows are thin system at t=-10ns, thin system at t=+12ns, thick system at t=-10ns, and thick system at t=+12ns. View is parallel to the axis of tip movement, the tip (centre) is not shown. Pleated P-beta conformations are shown in red, extended (AP-like) conformations are shown in blue. The thin system has collective AP-like conformation held together by axial hydrogen bonds, this is disrupted by indentation. The thick system has less AP-like conformation on its surface. Residues were coloured blue (for AP) where $\Phi < -120$ and $\Psi > 130$ degrees.
